# Highly Efficient Ru-Based Catalysts for Lactic Acid Conversion to Alanine

**DOI:** 10.3390/nano14030277

**Published:** 2024-01-29

**Authors:** Iunia Podolean, Mara Dogaru, Nicolae Cristian Guzo, Oana Adriana Petcuta, Elisabeth E. Jacobsen, Adela Nicolaev, Bogdan Cojocaru, Madalina Tudorache, Vasile I. Parvulescu, Simona M. Coman

**Affiliations:** 1 Department of Inorganic Chemistry, Organic Chemistry, Biochemistry and Catalysis, Faculty of Chemistry, University of Bucharest, 4-12 Regina Elisabeta Av., 030018 Bucharest, Romania; iunia.podolean@chimie.unibuc.ro (I.P.); mara.badea@s.unibuc.ro (M.D.); nicolae.guzo@s.unibuc.ro (N.C.G.); oana.petcuta@s.unibuc.ro (O.A.P.); bogdan.cojocaru@chimie.unibuc.ro (B.C.); madalina.sandulescu@chimie.unibuc.ro (M.T.); vasile.parvulescu@chimie.unibuc.ro (V.I.P.); 2 Department of Chemistry, Norwegian University of Science and Technology, Høgskoleringen 5, 7491 Trondheim, Norway; elisabeth.e.jacobsen@ntnu.no; 3 National Institute of Materials Physics, Atomistilor 405b, 077125 Magurele, Ilfov, Romania; adela.nicolaev@infim.ro

**Keywords:** ruthenium nanoparticles, MWCNT, beta-zeolite, magnetic nanoparticles, amination, lactic acid, alanine

## Abstract

The primary objective of this research was to develop efficient solid catalysts that can directly convert the lactic acid (LA) obtained from lignocellulosic biomass into alanine (AL) through a reductive amination process. To achieve this, various catalysts based on ruthenium were synthesized using different carriers such as multi-walled carbon nanotubes (MWCNTs), beta-zeolite, and magnetic nanoparticles (MNPs). Among these catalysts, Ru/MNP demonstrated a remarkable yield of 74.0% for alanine at a temperature of 200 °C. This yield was found to be superior not only to the Ru/CNT (55.7%) and Ru/BEA (6.6%) catalysts but also to most of the previously reported catalysts. The characterization of the catalysts and their catalytic results revealed that metallic ruthenium nanoparticles, which were highly dispersed on the external surface of the magnetic carrier, significantly enhanced the catalyst’s ability for dehydrogenation. Additionally, the -NH_2_ basic sites on the catalyst further facilitated the formation of alanine by promoting the adsorption of acidic reactants. Furthermore, the catalyst could be easily separated using an external magnetic field and exhibited the potential for multiple reuses without any significant loss in its catalytic performance. These practical advantages further enhance its appeal for applications in the reductive amination of lactic acid to alanine.

## 1. Introduction

As the basic building blocks of proteins synthesis, amino acids are used in nutrition and medicine fields, but the presence of reactive carboxylic acid and amine functionalities in their structure also made them valuable feedstocks for the synthesis of a myriad range of end products currently produced in the petrochemical industry [1].

Microbial cultivation processes are the primary method used for the production of amino acids [2,3]. However, these processes are expensive, time-consuming, and involve complex separation procedures [4]. Therefore, there is a strong interest in developing efficient synthetic chemical methods to convert abundant and renewable resources into amino acids. Unfortunately, such efforts have been largely unsuccessful thus far, with these methods relying on highly toxic and non-renewable chemical sources [5,6]. As an alternative, chemocatalytic approaches may provide a faster and potentially more efficient way to synthesize amino acids. However, the progress in this field has been hindered by the lack of easily achievable chemistry and catalytic materials.

Due to the accessibility to biomass-derived α-hydroxyl acids, their direct catalytic reductive amination with ammonia was the most envisaged route for obtaining α-amin acids [5,7,8]. Lactic acid (LA), easily accessible from biomass-derived substrates, possesses a hydroxyl group and a similar carbon block to alanine, which is qualified for alanine production through a reductive amination approach involving dehydrogenation, amination, and hydrogenation steps. However, until now, the reaction pathway was rarely discussed, but the amination reaction of alcohols to design an appropriate catalyst can be referenced [9]. Yan et al. [5] reported, for instance, a conversion of lactic acid to alanine with yields of around 43% at 220 °C over Ru/CNT catalysts, and this method was extended to other various biomass-derived α-hydroxyl acids, such as α-hydroxyl butyric acid, α-hydroxyl-3-methylbutyric acid, and α-hydroxyisocaproic acid. The yields to the corresponding α-amino acids were improved to 60–73% when ammonia and hydrogen were used by either modifying the pristine Ru/CNT catalyst with 10%Ni or by adding a homogeneous base (e.g., 1 mmol NaOH or KOH) as a promoter for the dehydrogenation step. A series of RuNi/AC (AC—activated carbon) catalysts was also reported by Chen and co-workers [10]. The authors achieved an optimal catalyst, with a content of 40%Ni and 60%Ru, leading to a yield of 64% to alanine, which is superior to the monometallic Ru/AC catalyst. Subsequently, Xie et al. [11] showed that the use of Ru/N-CNT catalysts promotes the synthesis of α-alanine (70% yield) from lactic acid under milder reaction temperatures (180 °C versus 220 °C).

Recently, Ma and co-workers [12] proposed polylactic acid (PLA) as an alternative lactic acid feedstock for alanine synthesis. The authors demonstrated that polylactic acid can be one-pot converted into alanine via ammonia treatment in the presence of a Ru/TiO_2_ catalyst. The process affords a 77% yield of alanine at 140 °C, and an overall selectivity of 94% can be reached by recycling experiments. Importantly, no additional hydrogen source was required, and the process could expand the application of polylactic acid waste and inspire new upcycling strategies for different plastic wastes.

Zeolites are efficient support materials for the stabilization and dispersion of metals or metal oxide active phases. Combined acid properties of zeolites with the redox properties of the metals lead to the generation of novel materials with improved catalytic properties in different applications for biomass conversion [13,14]. Related to the lactic acid amination toward α-alanine, only scarcely information on Ru/zeolite efficiency exists in the literature. Recently, Dusselier et al. [15] demonstrated the potential of Ru/beta catalysts for this synthesis, which were able to produce alanine with a selectivity of 80–86% and a lactic acid conversion of 24–55%, but the leaching and catalyst stability remains a concern.

Firstly, regarding the above-mentioned research on catalytic amination, it should be recognized that the conventional methods for separating catalysts, such as filtration and centrifugation, can be laborious and impede the complete separation of the catalyst. A promising solution to over-passing this issue is to anchor nanoparticles or homogeneous catalysts onto magnetic supports. In connection with this, Liu et al. [16] recently reported the development of a series of magnetically separable catalysts M/Ni@C (M = Ru, Pt, Pd, Ir, Rh) for the conversion of lactic acid to alanine. The Ru/Ni@C catalyst showed the best catalytic performance with a yield to alanine of 63.7%. Our research group also conducted significant research in the field of catalysts-based magnetic nanoparticles, and several studies on the catalytic efficiency of Ru-based magnetic nanoparticles (MNP) in different biomass upgrading processes are already reported in the literature [17,18].

Secondly, all catalysts use noble metals as active metal sites with a high catalytic efficiency in the dehydrogenation step, which is known as the rate-determining step in the amination of lactic acid. However, it is worth mentioning here that, irrespective of the catalysts design, Ru has superior catalytic performance over Pd, Pt, Rh, and Ir [5,19]. In addition, the presence of ruthenium oxide nanoparticles, acting as Lewis acid sites, alongside with metallic ruthenium nanoparticles could also activate the hydroxyl group by adsorption [20], while the use of the N-doped carrier (i.e., N-CNT) improves the dispersion of supported ruthenium nanoparticles and facilitates the adsorption of acidic reactants through basic sites [11].

Hence, combining the above-mentioned research findings, we report the high efficiency of Ru-based magnetic nanoparticle (MNP) materials in the catalytic amination of lactic acid to alanine. A comparison between the Ru/CNT and Ru/BEA catalysts developed in this work, and with other reported catalysts in the literature, indicates the superior catalytic performance of the Ru/MNP catalysts in terms of both lactic acid conversion and selectivity to alanine. The obtained results demonstrated that the efficiency of the catalytic process was significantly influenced by both the characteristics of the Ru-based catalysts and the reaction conditions.

## 2. Materials and Methods

All the chemicals and reagents were of analytical purity grade, purchased from Sigma-Aldrich, and used without further purification. The multi-walled carbon nanotubes (MWCNTs) were purchased with the following features: preparation method—Catalytic Chemical Vapor Deposition (CVD) (CoMoCAT^®^); over 95% carbon; O.D × L/6–9 nm × 5 μm; and armchair configuration. Hydrated ruthenium chloride (RuCl_3_·3 H_2_O) had ~37% Ru basis. PTFE membrane filters with 0.45 μm pore size were purchased from Merck.

### 2.1. Catalysts’ Synthesis

Ru supported onto MWCNT (Ru/CNT): The MWCNT-supported Ru nanoparticles were synthesized by wet impregnation method. The Ru loading (i.e., 1 wt%, 3 wt% and 5 wt%) was adjusted by controlling the amounts of MWCNT powder and the aqueous solution of RuCl_3_·3 H_2_O. Typically, for the synthesis of 1%Ru/CNT sample, MWCNT (1.0 g) was added into the aqueous solution of RuCl_3_·3 H_2_O (26.1 mg in 10 mL water) and was then subjected to stirring for 1 h at room temperature. The solution was aged for 2 h. Afterward, the water was evaporated at 80 °C, under vacuum. The catalyst was then calcined at 350 °C, in static atmosphere, for 4 h, and reduced in H_2_ gas at 450 °C for 6 h. The obtained samples were denoted as 1%Ru/CNT, 3%Ru/CNT, and 5%Ru/CNT.

Ru supported onto beta-zeolite (Ru/BEA): Ru-based catalysts were prepared by the deposition–precipitation (DP) of RuCl_3_·3 H_2_O onto a BEA zeolite with Si/Al ratio of 12.5. In a typical preparation approach, for the synthesis of the 1 wt% Ru/BEA catalyst, a solution of 26.1 mg RuCl_3_·3 H_2_O (0.1 mmol of RuCl_3_·3 H_2_O in 60 mL H_2_O) was dropwise added to a suspension of zeolite (1 g in 80 mL of water) under stirring. Afterward, a solution of sodium hydroxide (0.1 M) was slowly dropwise added until a pH of 10. The mixture was further stirred for 24 h at room temperature. After deposition, the obtained solid was separated from the liquid phase by centrifugation (6000 rpm for 35–40 min) and washed until a neutral pH and until no chlorine anion was detected in the rinse water with an AgNO_3_ reagent. After washing, the catalysts were dried under vacuum at 110 °C, for 2 h, calcined at 300 °C for 4 h, and reduced at 450 °C under a hydrogen flow (30 mL/min) for 6 h (heating rate—1 °C /min). The obtained samples were denoted as 1%Ru/BEA and 3%Ru/BEA.

Ru supported onto magnetic nanoparticles (Ru/MNPs): The catalysts were produced by applying a previously reported procedure in the literature [18]. The approach involved four steps: (i) The synthesis of magnetite nanoparticles (MNP) through the base-assisted co-precipitation of a FeCl_2_·4 H_2_O and Fe(NO_3_)_3_·9 H_2_O mixture. For this, 0.8 g FeCl_2_·4 H_2_O was added to an aqueous solution of 3.23 g Fe(NO_3_)_3_·9 H_2_O in 80 mL degassed water. The mixture was stirred at 90 °C, under Ar atmosphere, for 30 min. To the orange solution formed, 6 mL of NH_4_OH (25%) was rapidly added under vigorous continuous stirring until a black, magnetic precipitate was formed. The black suspension was further stirred for another 2 h at 90 °C under inert atmosphere; then, the nanoparticles (NPMs) were magnetically separated using an external magnet and washed with ethanol. (ii) The silica coating of MNPs by using tetraethyl orthosilicate (TEOS). The MNPs were further redispersed in water in the ultrasonic bath and NH_4_OH (25%) was dropwise added until a pH of 9–11 was achieved. Subsequently, 3.5 mL of tetraethyl orthosilicate (TEOS) was added and the mixture was stirred at 40 °C, overnight. The obtained silica-coated particles were washed with water and ethanol, then dried for 24 h, at 80 °C. (iii) Functionalization of the silica shell through the silanization with (3-aminopropyl) triethoxysilane (APTES) in anhydrous toluene for 24 h, at 80 °C. After magnetic separation, the functionalized nanoparticles were washed again with water and ethanol, then dried for 24 h, at 80 °C. (iv) Ru/MNP catalysts were prepared by the deposition–precipitation (DP) of RuCl_3_·3 H_2_O onto the APTES silica-coated MNP carrier at a basic pH (pH = 13) ensured by a solution of NH_4_OH (25%). The prepared catalysts were dried at 80 °C and subsequently calcined at 120 °C, for 3 h. The obtained materials were further reduced with sodium borohydride (10 mg NaBH_4_ for 100 mg of catalytic sample) in a mixture of 20 mL ethanol with 20 mL water, at room temperature, and for 12 h. The obtained samples were further magnetically separated, dried at 80 °C, and subsequently calcined at 120 °C, for 3 h. The loading of the ruthenium precursor corresponds to a concentration of 1 wt% and 5 wt% Ru. For comparison, part of the sample with 5 wt% Ru was also used in an unreduced form, as Ru(III)/MNP.

**Scheme 1:**
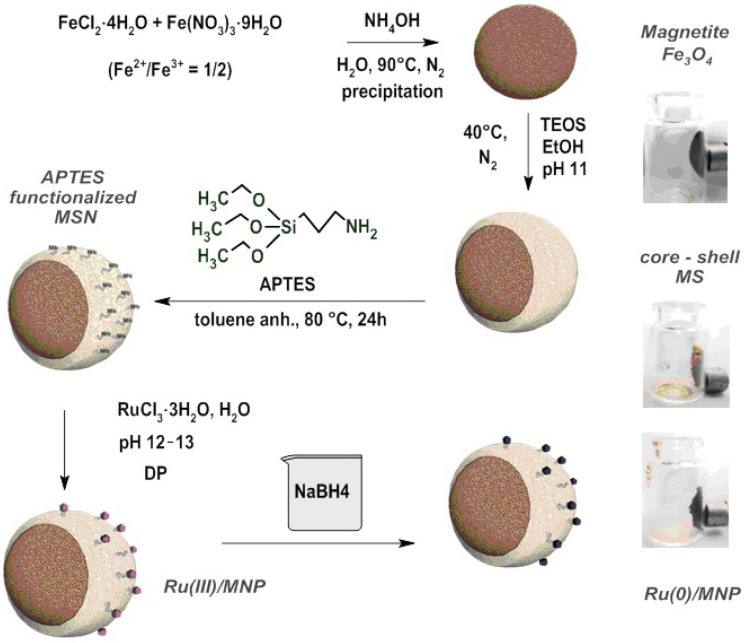
General synthetic scheme for the preparation of Ru(0)/MNP and Ru(III)/MNP samples.

The general basic synthetic strategy used in this work for the anchoring of ruthenium active species on magnetic support is illustrated in Scheme 1.

The obtained samples were denoted as 1%Ru(0)/MNP, 5%Ru(0)/MNP, and 5%Ru(III)/MNP.

### 2.2. Catalysts’ Characterization

Prepared samples were characterized by techniques as adsorption–desorption isotherms of liquid nitrogen at −196 °C, X-ray diffraction (XRD), temperature programmed desorption of H_2_ and NH_3_ (H_2_-TPD and NH_3_-TPD), IR diffuse reflectance with Fourier transform (DRIFT) spectroscopy, scanning transmission electron microscopy (STEM), and X-ray photoelectron spectroscopy (XPS).

Surface area, pore volume, and pore diameter were determined from the adsorption–desorption isotherms of nitrogen at −196 °C using a Micromeritics ASAP 2020 Surface Area and Porosity Analyzer. The specific surface area (S_BET_) was calculated following the BET (Brunauer–Emmett–Teller) procedure with eight relative pressures (p/p_0_) of nitrogen in the range of 0.07–0.20. The t-plot method was used to determine the micropore and external surface area and also the micropore volume. The Barret–Joyner–Halenda (BJH) method was used to determine pore size distribution, considering the desorption curves. Powder X-ray diffraction patterns were collected at room temperature using a Shimadzu XRD-7000 apparatus with the Cu Kα monochromatic radiation of 1.5406 Å, 40 kV, and 40 mA at a scanning rate of 1.0 2θ/min, in the 2θ range of 5°–90°. Hydrogen and NH_3_- temperature-programmed desorption (H_2_- and NH_3_-TPD) values were recorded by using a Micromeritics apparatus—Autochem II (Chemisorption Analyzer). Approximately 20 mg of freshly reduced sample was heated at 650 °C under N_2_ for 0.5 h to remove the hydrogen adsorbed on Ru atoms. After that, the temperature was reduced to 150 °C and until baseline became stable. Subsequently, successive doses of H_2_ gas (H_2_-TPD) or NH_3_ gas (NH_3_-TPD) were provided. DRIFT spectra were recorded with a Thermo 4700 spectrometer (400 scans with a resolution of 4 cm^−1^) in the range of 400–4000 cm^−1^. The morphology of the ruthenium particles deposited on MWCNTs was analyzed by using a scanning transmission electron microscopy (STEM) system. Bright-field scanning transmission electron microscopy (BF-STEM) and dark-field scanning transmission electron microscopy (DF-STEM) images were collected from Hitachi S-5500 operating at 30 kV accelerating voltage. Bright-field and dark-field images were collected at different resolutions in order to increase the clarity of the dispersion of metals onto the external surface of the carbon nanotubes. The average diameter of the particle was calculated using a graphical method, considering minimum of 10 ruthenium nanoparticles. The X-ray photoelectron spectroscopy (XPS) analysis of the sample was performed in an AXIS Ultra DLD (Kratos Surface Analysis) setup using Al Kα1 (1486.74 eV) radiation produced by a monochromatized X-ray source at operating power of 144 W (12 kV × 12 mA). The base pressure in the analysis chamber was around 1 × 10^−9^ mbar. The XPS investigation was carried out to determine the chemical composition of the sample. All core level spectra were deconvoluted with use of Voigt functions, singlets, or doublets (Lorentzian and Gaussian widths) with a distinct inelastic background for each component [21,22]. The minimum number of components is used to obtain a convenient fit. The binding energy scale was calibrated to the C 1 s standard value of 284.6 eV (measured at the beginning of XPS spectra).

### 2.3. Catalytic Tests

The catalytic experiments were carried out in a stainless steel autoclave (15 mL, HEL Instruments). Briefly, to a solution of lactic acid (in amounts of 31.5 mg (0.35 mmol), 63 mg (0.7 mmol) and 500 mg (5.6 mmol)) in 5.0 mL NH_3_·H_2_O (solution of 28 wt%), 25 mg of catalyst were added. The resulting mixture was stirred at 180–220 °C, under 10 atm of H_2_, and for 0.5–8 h. For comparison, a commercial sample of 5%Ru/C, purchased from Johnson Matthey, was also tested. A blank reaction in which a solution of 25 mg (0.27 mmol) of lactic acid in 5 mL NH_3_·H_2_O (solution of 28 wt%) was maintained for 2 h under stirring at 200 °C, at a hydrogen pressure of 10 atm, was also performed.

After the reaction, the autoclave was quickly cooled at room temperature, the catalyst was recovered by centrifugation, and the products were separated by solvent distillation under vacuum.

### 2.4. Products’ Analysis

The recovered products were silylated with 200 μL of a derivatization agent N-Methyl-N-tert-butyldimethylsilyltrifluoroacetamide (MTBSTFA) [23,24] (Scheme S1) in 200 μL of pyridine, at 80 °C for 4 h, diluted with 1 mL of ethyl acetate, and analyzed with a GC-MS THERMO Electron Corporation instrument equipped with TG-5SilMS column 30 m × 0.25 mm × 0.25 μm. The injector port was set up at 230 °C. The temperature in the oven was kept at 50 °C for 5 min and then increased to 250 °C at a rate of 10 °C/min at a pressure of 0.38 Torr with He as the carrier gas. More details on the products’ analysis are provided in Supplementary Materials (Figures S1–S4).

Potentially carboxylic acid by-products (e.g., acetic and pyruvic acids) and untransformed lactic acid were also analyzed by liquid chromatography with a HPLC-DAD/RID system from Agilent equipped with a ZORBAX carbohydrate column in the following conditions: mobile phase—3 mM H_2_SO_4_ in water (0.294 g/1 L); mobile phase flow rate—0.5 mL/min; pressure—98 bar; injection volume—10 µL; and run time—30 min. Detection was performed with DAD (210 nm) detector. The analyzed dry samples were mixed with the mobile phase until complete dissolution without further treatment.

The conversion of lactic acid (*X*) and selectivity to reaction products *n* (*S_n_*) were calculated from the chromatographic analysis by using the follow equations:

$$X\%\;=\frac{n_{i}\;-\;n{\;}_{t}\;-\;}{n_{i}}\;\;\times \;100S_{n}\%\;=\;\frac{Yield_{n}}{X}\;\times \;100$$

where *n_i_*—initial moles of lactic acid; *n_t_*—moles of untransformed lactic acid at time “*t*”, determined from chromatographic analysis.

## 3. Results and Discussion

### 3.1. Catalysts’ Characterization

#### 3.1.1. Ruthenium-Based MWCNT Samples

The textural characteristics of the Ru/CNT samples, as obtained through BET, t-plot, and BJH techniques, are presented in Table 1. Additionally, the nitrogen adsorption–desorption isotherms at −196 °C, along with the corresponding distribution of pore sizes, are presented in Figures S5–S7.

**Figure 1:**
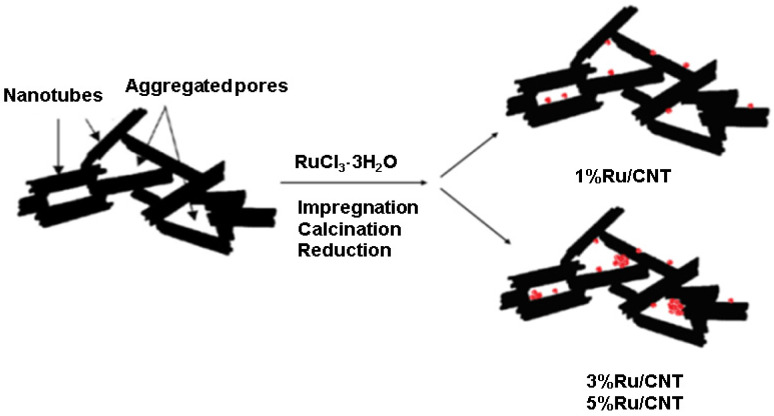
Schematic structural model for the aggregated pores in MWCNTs and the structural models of the Ru/CNT samples.

The adsorption–desorption isotherms (Figures S5–S7) exhibit characteristics of Type IV isotherms. At a relative pressure of 0.01 (p/p_0_), there is only a slight increase in nitrogen adsorption, indicating a low surface area and volume of micropores (as shown in Table 1, columns 4 and 7). The formation of a surface monolayer is evident within the range of p/p_0_ = 0.01–0.4, while the hysteresis loop associated with capillary condensation in mesopores is observed at a medium relative pressure range (p/p_0_ = 0.4–0.85) [25]. This can be attributed to the presence of small mesopores with a diameter of 3.3 nm (Table 1, column 9), which is similar to the inner cavity diameter of the pristine MWCNT [26]. The data in Table 1 confirm that this diameter remains constant regardless of the ruthenium loads, indicating that there is no deposition of ruthenium in the inner cavity of the MWCNT during the impregnation process. As the pressure approaches the saturation pressure (p/p_0_ = 0.85–0.99), the adsorption amount significantly increases, indicating a strong capillarity in larger mesopores, ranging from 27.7 to 31.9 nm (Figures S5–S7 and Table 1, column 9). According to Cheng and co-workers [26], such hysteresis loops of H3 type correspond to pores approximately 20–40 nm in size in MWCNT, which are likely formed by the confined space among the isolated nanotubes with different orientations, and interact through inter-molecular forces. These interactions create a relatively stable aggregated structure (Figure 1).

**Figure 2:**
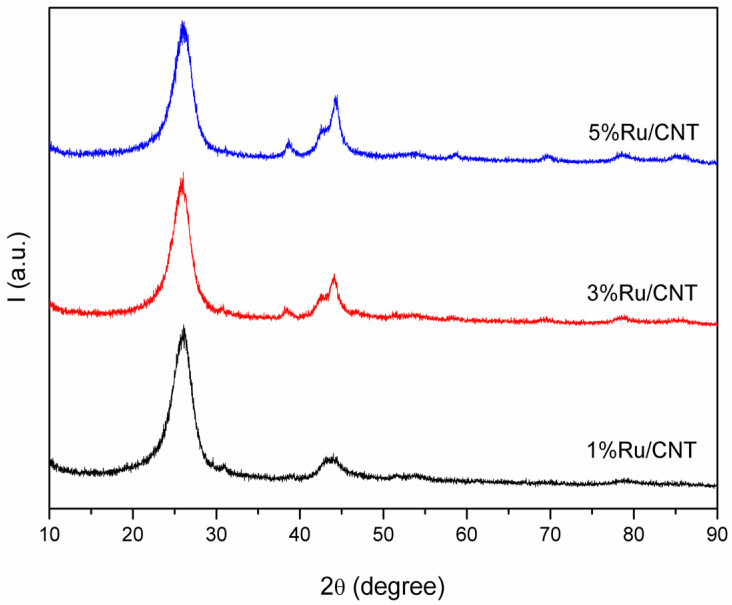
XRD patterns of the Ru-based MWCNTs catalysts.

The constant inner cavity diameter, combined with the reduction in the external surface area as the ruthenium load increases, clearly indicates the deposition of ruthenium on the outer surface of the MWCNT tubes. The coalescence of the Ru particles on the external surfaces of MWCNTs with increased ruthenium loadings (specifically, 3% and 5%) is further supported through XRD and STEM analyses. The examination of the XRD patterns (Figure 2) reveals that MWCNT exhibits two distinct reflection peaks at 26° and 43.9°, aligning with the (002) and (100) facets, respectively, which is in accordance with the previously reported data [27,28]. The structure of MWCNTs remains undamaged during the impregnation process with varying quantities of ruthenium salts and subsequent activation steps. Furthermore, there is no indication of additional diffraction lines associated with ruthenium particles in the 1%Ru/CNT sample, implying the formation of well-dispersed nanoparticles with a narrow size range. This observation is illustrated in Figure 2 (pattern in black).

However, in the case of samples containing 3%Ru and 5%Ru, in addition to the distinctive lines of the MWCNT carrier, there are some identifiable lines at 38.3°, 42.8°, 58.4°, 69.6°, and 78.7° (corresponding to the (100), (101), (102), (110), and (103) reflections) which can be attributed to the metallic Ru (specifically in its hexagonal phase; as documented in JCPDS Card No. 06–0663) [29]. This suggests an agglomeration of the ruthenium species during the preparation of the samples, resulting in the formation of larger ruthenium particles. The average size of ruthenium crystallites was found to be 9.0 nm for the 3%Ru/CNT sample and 10.6 nm for the 5%Ru/CNT sample, calculated using the Debye–Scherrer equation [30], by considering the (100) reflection of the ruthenium particles.

**Figure 3:**
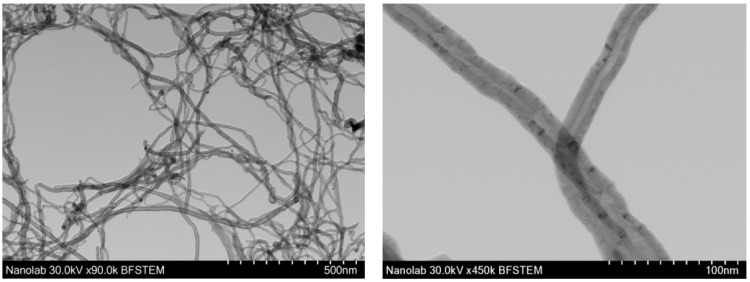
BF-STEM images for the 1%Ru/CNT sample.

The images obtained through STEM microscopy indicate that the ruthenium particles are deposited on the outer surface of the carbon nanotubes. In the case of the 1%Ru/CNT sample, the STEM microscopy analysis revealed an even distribution of tiny ruthenium particles, each measuring an average size of 2.5 nm (Figure 3). This observation aligns with the findings from the XRD analysis. Additionally, the distribution of these particles suggests a slight agglomeration in the case of the 3%Ru/CNT sample and 5%Ru/CNT sample (Figures S8 and S9) with an average particle size of 5.0 nm and 7.0 nm, respectively.

**Figure 4:**
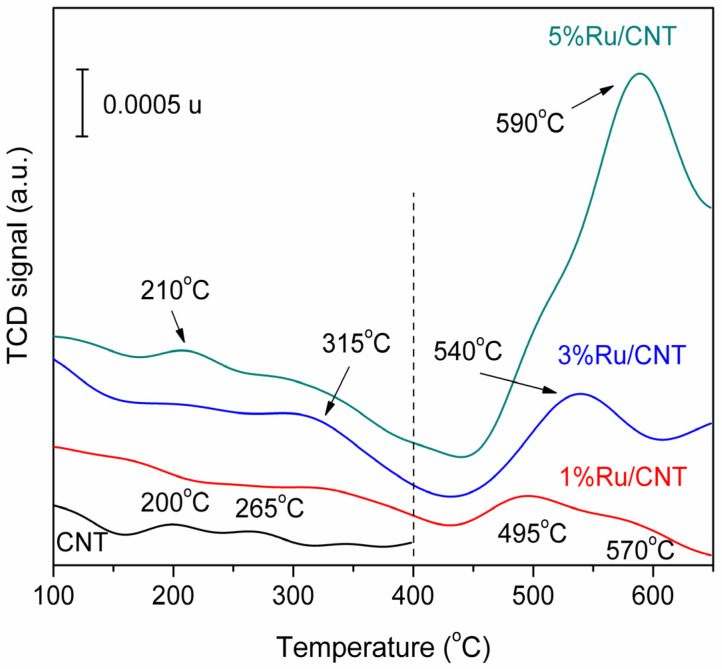
NH_3_-TPD profiles for the Ru/CNT samples.

As anticipated, the carrier of multi-walled carbon nanotubes (MWCNTs) comprises minimal acid sites. The NH_3_-TPD analysis results shows that these acidic sites are predominantly located in the low temperature (LT) range, specifically below 400 °C (Figure 4, illustrated by the black line).

However, the impregnation of ruthenium chloride followed by calcination and reduction lead to both Lewis and Brønsted acid sites in the high temperature region (above 400 °C). Nevertheless, the concentration level of these sites is very low regardless of the increasing ruthenium loads, varying from 0.4 μmol/g (1%Ru/CNT) to 2.2 μmol/g (5%Ru/CNT). It is worth noting that the larger ruthenium oxide particles are more resistant to reduction, and the appearance of new peaks may be attributed to the presence of partially reduced RuO_x_-OH species in addition to the metallic particles for samples with 3 wt% and 5 wt%Ru (Figure 4) [31].

Temperature-programmed desorption of hydrogen (H_2_-TPD) brought additional information about these catalysts (Figure S10). The MWCNT carrier showed no measurable peaks of hydrogen, indicating a low binding energy of hydrogen adsorption [32]. This is generally attributed to the outer surface curvature, where hydrogen molecules primarily adsorb [33]. For the Ru/CNT catalysts, the desorption was rather small. It started at around 200 °C with a variation in the maxima depending on the ruthenium loading (Figure S10). However, the recorded profiles suggest a diversity in the strength of the sites, where hydrogen is adsorbed in a dissociative way, and the presence of spillover hydrogen. The chemisorbed hydrogen varied in the following order: 0.026 mmol/g (1%Ru/CNT) < 0.038 mmol/g (3%Ru/CNT) < 0.046 mmol/g (5%Ru/CNT). This order corresponded to the stoichiometry of the following order: 0.57 × 10^21^ molecules of hydrogen/g Ru (5%Ru/CNT) < 0.76 × 10^21^ molecules of hydrogen/g Ru (3%Ru/CNT) < 1.56 × 10^21^ molecules of hydrogen/g Ru (1%Ru/CNT). Thus, this confirmed that there was a decrease in the dispersion of the active metal with metal loading. Furthermore, the H_2_-TPD for the 3% and 5% Ru/CNT catalysts showed additional desorption peaks at above 500 °C, indicating a stronger chemisorption of hydrogen on these catalysts [34].

**Figure 5:**
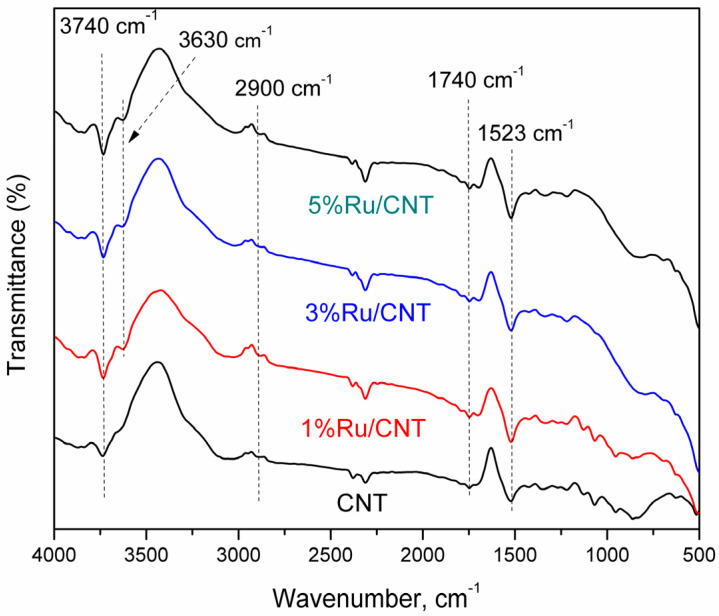
DRIFT spectra of the MWCNT and Ru/CNT samples.

Figure 5 presents the infrared spectra for the MWCNT and Ru/CNT samples. The infrared spectrum of the pristine MWCNT closely resembles those previously reported in the literature [35]. However, it is important to note that observed peaks primarily arise from defects and impurities rather than the inherent structure of the MWCNT itself. The IR spectrum of pristine CNT exhibits characteristic peaks related to O−H vibrations at 3740 cm^−1^, C−H stretching vibrations at approximately 3000 cm^−1^, carboxylic groups at 1740 cm^−1^, and −C=C− stretching vibrations at 1523 cm^−1^. The 1000–1300 cm^−1^ region may be assigned to the O−O vibration mode of ester, ether, phenol, or carboxyl groups.

The IR analysis revealed similar peaks in all Ru/CNT samples. Additionally, a distinct peak was detected at 3630 cm^−1^, which could potentially be linked to the vibrations of O−H bonds in RuO_x_−OH clusters.

#### 3.1.2. Ruthenium-Based BEA Zeolite Samples

The deposition–precipitation (DP) method has been widely used for the synthesis of nanostructured materials. In this approach, hydroxide ions are gradually resealed and the metal salts precipitate homogeneously avoids the formation of large metal nanoparticles [36]. Therefore, compared with direct impregnation, the DP method could provide the way to prepare uniformly dispersed metal nanoparticle catalysts and improve the ability of metal dispersion [37]. However, during the DP process, the crystallinity of the zeolites could be partially affected, even if the process is carried out at room temperature. In connection with this, Groen et al. [38] showed that, compared with other zeolite topologies, beta-zeolite suffers an easier Si extraction in a basic medium, probably as a result of the less stable framework and the relatively large interconnected channels. However, among zeolites from the same family (i.e., beta-zeolite in this work), the desilication behavior is also a consequence of the different zeolite framework Si/Al ratio, which influences the kinetics of Si extraction. The framework desilication is hindered at a low Si/Al ratio as a result of the relatively high concentration of Al (i.e., the negatively charged AlO_4_^−^ tetrahedrons), which creates a more stable framework for silicon extraction. In this case, the hydrolysis of the Si-O-Al bond in the presence of OH^-^ is hindered compared to the relatively easy cleavage of the Si-O-Si linkage in the absence of neighboring tetrahedral Al [38].

**Figure 6:**
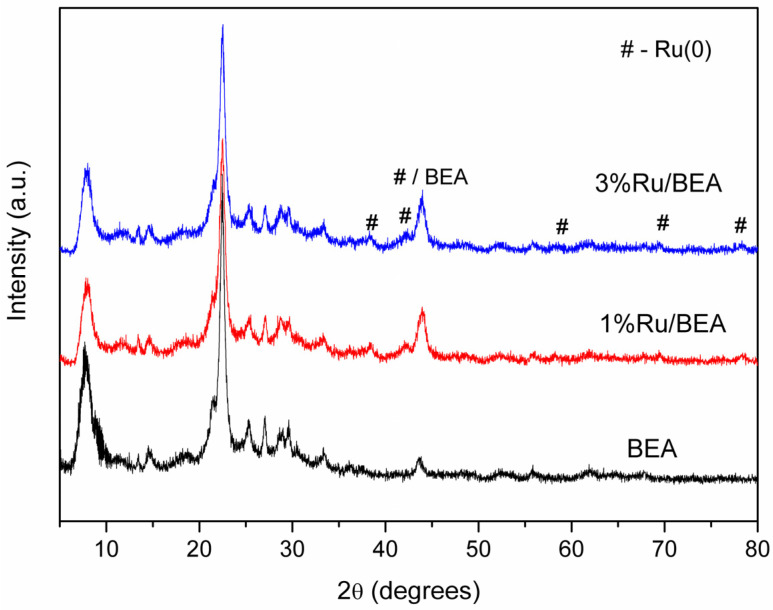
XRD patterns for pristine zeolite and Ru/BEA samples.

The XRD patterns of the Ru/BEA samples confirm the above findings, with the presence of the characteristic diffraction lines (2θ of 7.6, 21.2, and 22.5°) of the pristine zeolite in the Ru/BEA patterns indicating a preservation of the crystalline structure of BEA topology (Figure 6) [29,39].

The XRD patterns evidenced new reflection lines at 38.5, 42.3, 44.1, 58.4, 69.6, and 78.5° in the Ru/BEA samples, which are assigned to the (100), (002), (101), (102), (110), and (103) planes of bulk hexagonal Ru metal (ICDD-JCPDS Card No. 06-0663) [29] but do not evidence the reflection lines characteristic to RuO_2_ (i.e., the lines at around 28.1, 35.1, 44.0, and 54.4°, indexed to the (110), (101), (111), and (211) planes of anhydrous crystalline RuO_2_ (ICDD-JCPDS Card No. 43-1027)). This indicates a higher dispersion of the latter. However, the size of the metallic ruthenium particles is difficult to calculate because of the ambiguous boundary of the characteristic diffraction lines.

**Figure 7:**
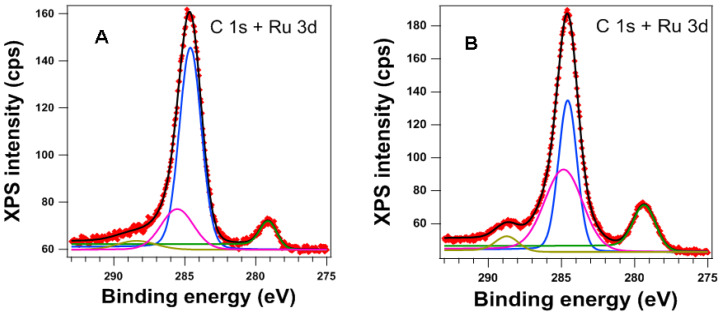
The XPS Ru3d spectrum of 1%Ru/BEA (**A**) and 3%Ru/BEA (**B**) samples.

On the other hand, the Ru3d spectra showed two bands at 279.1 eV, indicating the presence of Ru metal [40], and a second one from Ru3d_3/2_, at 284.2/285.6 eV, indicating the presence of RuO_x_ (Ru^x+^) species (Figure 7). The XPS spectrum in the Ru3d region is complex due to the overlapping of the C1s signal (284.6 eV) and Ru3d doublet (5/2 and 3/2). Therefore, the spin-orbit splitted Ru3d doublet (Ru 3d_5/2_ and Ru 3d_3/2_) was resolved by applying a set of narrow (0.6–0.8 eV) symmetric components in agreement with Balcerzak et al. [40].

The Ru^x+^/Ru^0^ ratios were calculated using the area% of the Ru^x+^ and Ru^0^ peaks. Interestingly enough, irrespective of the Ru loads, the concentration of the RuO_x_ species predominates, with the Ru^x+^/Ru^0^ ratio slightly varying from 3.0 (1%Ru/BEA, 25.06%Ru(0) and 74.94%RuO_x_) to 3.2 (3%Ru/BEA, 23.87% Ru(0) and 76.13% RuO_x_). The direct impregnation approach lead to larger ruthenium particles and a larger degree of reduction, and the metal particle size varied according to the depth of the metal penetration in the zeolite pores [41]. Meanwhile, during the DP preparation applied in this work, high dispersed RuO_x_ species—not detectable in XRD patterns—were formed on the surface of the beta-zeolite alongside with uniformly dispersed small metallic ruthenium particles. Moreover, no chlorine and sodium XPS peaks could be found in the samples, even after prolonged accumulation times.

**Figure 8:**
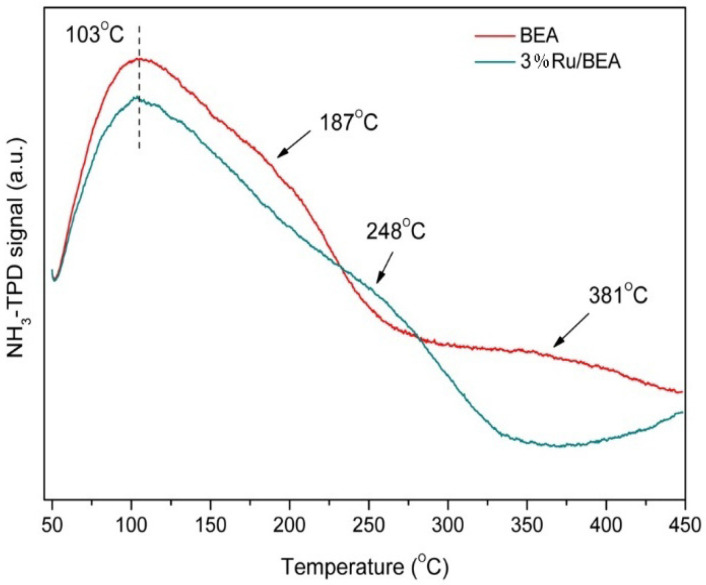
NH_3_-TPD profiles for pristine BEA zeolite and 3%Ru/BEA sample.

The NH_3_-TPD measurements (Figure 8 and Table S2) confirmed the existence of the acid sites with different strength. The desorption process occurring in the range of 50–200 °C corresponded to the weak acid sites. In the case of the beta-zeolite, this peak was observed to consist of two components, with maximum values at 103 °C and 187 °C. Furthermore, there was an additional peak at 381 °C, indicating the presence of acid sites with medium strength. In agreement with Serrano and co-workers [42], peaks centered at low temperatures (<200 °C) (Figure 8) were conventionally associated with ammonia molecules adsorbed onto weak acid sites, such as silanol groups, while those centered at 350–400 °C were related to the interaction of ammonia molecules with the framework aluminum species. Following the deposition of ruthenium, modifications in the NH_3_-TPD profile indicated a change in acidity. The deposition of Ru on the zeolite surface can occur through various mechanisms, involving both Al sites and framework silanols. The NH_3_-TPD profile of the Ru/BEA reveals the presence of both types of interactions (Figure 8). The disappearance of the peak centered at 381 °C confirms that the deposition of ruthenium takes place with the involvement of these medium-strength acid sites. The new maxima detected in the NH_3_-TPD profile of Ru/BEA, centered at 248 °C, can be attributed to the presence of the RuO_x_ crystallites, which is consistent with the XPS results [42].

The N_2_ adsorption–desorption isotherms of the pristine BEA and Ru/BEA zeolites are presented in Figure S11. The isotherms exhibit a combination of Type I and Type IV behavior, accompanied by a Type 3 (H3) hysteresis loop—according to the IUPAC classification. This indicates the presence of micropore filling at low pressures (p/p_0_ < 0.1) and hysteresis loops at higher pressures (p/p_0_ of 0.65–0.99), illustrating a hierarchical porous system comprising both micro- and mesoporosity [43].

According to the data presented in Table 2, the deposition of ruthenium using the DP approach resulted in a reduction in the overall surface area (BET surface, Table 2, column 3). Furthermore, the micropore surface area (Table 2, column 5) experienced a more substantial decline compared to the external surface (Table 2, column 4). This observed difference can be attributed to the predominant deposition of ruthenium species on the inner surface of the narrow zeolite pores. However, it should be noted that the catalysts exhibit a high Langmuir and external (t-plot) surface area, indicating that the pores of the zeolite were not blocked.

#### 3.1.3. Characterization of Ru/MNP Samples

**Figure 9:**
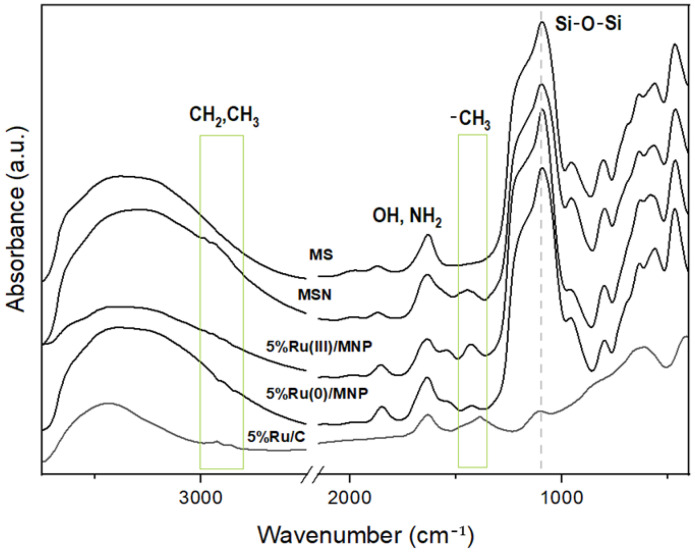
DRIFT spectra of the magnetic-based samples in comparison to the commercial Ru/C sample.

The successful deposition of the successive layers (i.e., silica—MS and APTES—MSN) and ruthenium species on the MNP surface can be easily demonstrated with the DRIFT measurements [18]. The DRIFT spectrum displays two distinct peaks at 640 and 560 cm^−1^, which are specific to Fe−O−Si moieties (Figure 9). Additionally, there are other visible bands at 1100, 960, and 800 cm^−1^, indicating the presence of Si−O−H and Si−O−Si groups. The bands in the range of 1490–1450 cm^−1^ are linked to the presence of free −NH_2_ and −CH_2_ groups, while the band at 1640 cm^−1^ corresponds to the OH groups from the water adsorbed on the solid surface (H−OH stretching vibrations), which overlaps with the distinctive band of the free amine [44]. The broad band observed at around 3500 cm^−1^ can be attributed to the vibration of the O−H bond, but it may also indicate the presence of N−H, leading to a shift in the absorption maximum after functionalization with APTES. Following the functionalization with APTES, a new band emerges at 1450 cm^−1^, which can be attributed to the deformation vibrations of the −CH_3_ group in the ethoxy region of APTES. Additionally, the presence of the propyl group is further confirmed by the characteristic bands of the −C−H bond vibration at 2945 and 2862 cm^−1^ [45].

The peaks observed in the DRIFT spectrum of the commercial Ru/C catalyst predominantly originate from adsorbed water (Figure 9). The presence of the precursor RuCl_3_ causes the emergence of a band at 1540 cm^−1^, which was formerly associated with the −C−NH_3_^+^Cl^−^ entity [18]. Our previous EXAFS, XPS, and EDX analyses on 5%Ru(III)/MNP sample also confirmed that the reduced number of chlorine neighbors remained as adatoms of Ru [18].

**Figure 10:**
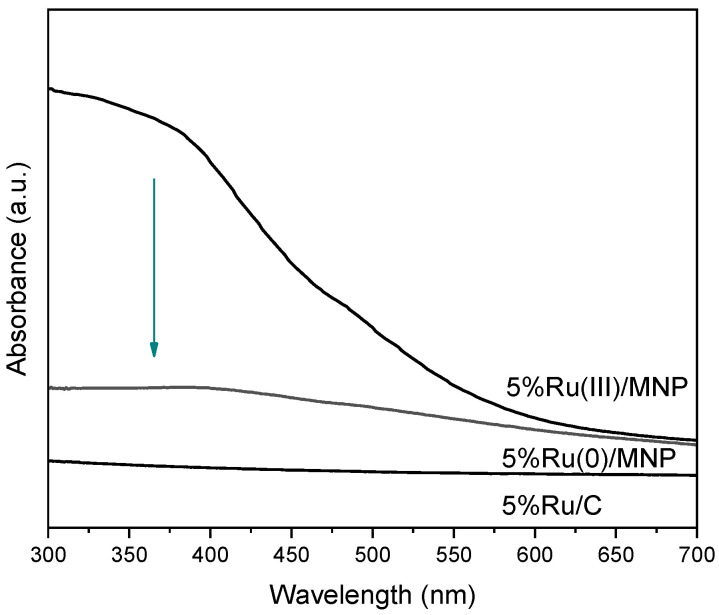
The UV-vis spectra of Ru(III)/MNP and Ru(0)/MNP samples in comparison to the commercial Ru/C sample.

The intensity of the peak associated with the −C−NH_3_^+^Cl^−^ entity highly decreased as a consequence of the reduction process using NaBH_4_ (Figure 9). The reduction of docked ruthenium species was also evidenced by UV-vis spectra (Figure 10). In accordance with a previous work [41], the formation of Ru(OH)_x_Cl_3−x_ species takes place at the beginning of the process, which further interacts with the amino groups of the magnetic nanoparticles where they are docked. This interaction is evidenced by a wide absorbance peak at 407 cm^−1^, owing to the transfer of charge between the metal and ligand (Figure 10) [17]. After sample treatment with NaBH_4_, this peak almost entirely vanishes, thereby confirming the high conversion of the ruthenium precursor into metallic species.

**Figure 11:**
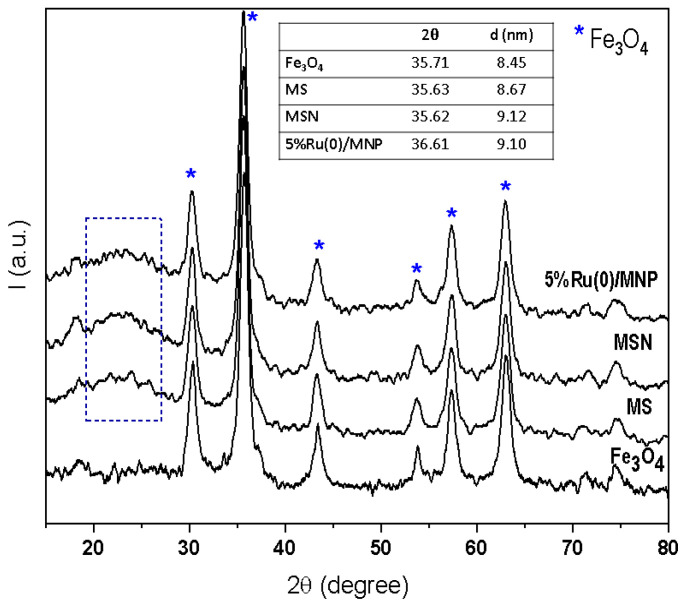
The XRD patterns of 5%Ru(0)/MNP sample in comparison to magnetite, core–shell MS, and functionalized core–shell MS (MSN) (inset: the average size of the crystallites calculated with Scherrer formula at corresponding angle of the strongest reflection (311)).

The X-ray diffraction results (Figure 11) reveal that the synthesized MNP primarily consists of magnetite in its oxide form. The diffraction patterns display distinct lines at angles of 2θ at 30.1°, 35.4°, 43.1°, 53.4°, 57.1°, and 62.6°, which correspond to the unique crystal planes of cubic magnetite ((220), (311), (400), (422), (511), and (440), JCPDS 19-629) (indicated in XRD patterns with an asterisk (*)) [18]. No diffraction lines specific to hematite (α-Fe_2_O_3_, usually formed under thermal dehydration conditions) or goethite (α-FeOOH, usually formed by hydrolysis reaction) were observed in the XRD pattern. After coating the magnetite with silica, a broad diffraction line at 2θ of around 22° becomes visible, indicating the presence of an amorphous silica layer (marked with a rectangle in Figure 11). The XRD pattern of the Ru(0)/MNP sample does not show any other diffraction lines, suggesting that the ruthenium species are well-dispersed on the surface of the magnetic nanoparticles.

The Scherrer formula [30] was used to estimate the average size of the crystallites and the obtained values are given in the inset of Figure 11. The increase in the crystallite size with successive functionalization suggests the formation of a narrow layer of silica and APTES on the surface of the magnetite [18].

In sum, the catalysts’ characterization evidenced the presence of oxide ruthenium and metallic ruthenium nanoparticles on both Ru/CNT and Ru/BEA catalysts, while Ru/MNP mostly contained metallic ruthenium nanoparticles on the external surface of the carrier coupled with -NH_2_ basic sites provided by the APTES species. The presence of a high density of weak acid sites of the zeolite carrier (Ru/BEA catalyst) should also be added to this picture. The influence on the catalytic performances of these different characteristics will be discussed in the following discussion.

### 3.2. Catalytic Tests

In order to confirm the catalytic character of the alanine synthesis, this study started with blank experiments. Therefore, in the presence of the ammonia solution, the lactic acid was converted to lactamide, irrespective of the presence or the absence of hydrogen. The increase in the temperature, from 180 to 200 °C, lead to a slightly increase in lactic acid conversion (from 10.2% to 12.6%) with a total selectivity to lactamide. These results confirm the findings of Tian et al. [12], which reported that lactamide is formed under heating conditions from the neutralization reaction of the lactic acid with ammonia to ammonium lactate followed by its dehydration toward lactamide. The Ru-based catalysts influenced the products’ distribution, being essential for the dehydrogenation/rehydrogenation and amination steps, as the catalytic results demonstrate (Table 3).

At a low load of lactic acid (i.e., 0.35 mmol, Table 3, entry 2), a yield of 70.5% to alanine was obtained in the presence of the 5%Ru(0)/MNP catalyst, which is 1.3 times higher than that on the 5%Ru/CNT (55.7%, Table 3, entry 1), while the Ru/BEA catalysts mainly directed the reaction toward the alanine isomer, namely lactamide. Increased lactic acid loads (i.e., 0.7–5.6 mmol) highly decreased the yield to alanine, irrespective of the catalyst’s nature.

**Scheme 2:**
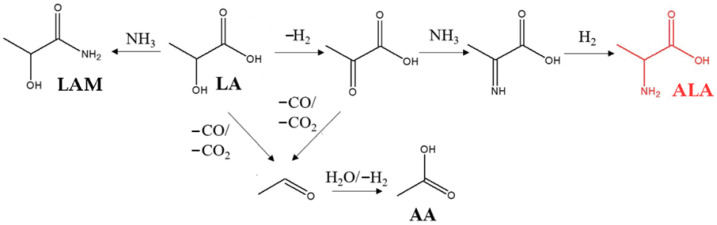
Reactions pathways of lactic acid (LA) conversion to lactamide (LAM), alanine (ALA), and acetic acid (AA).

**Figure 12:**
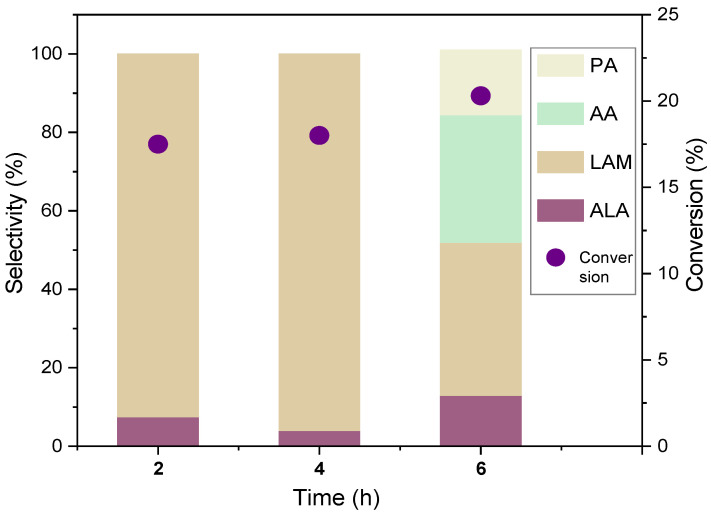
Distribution of reaction products at different reaction time in the presence of 5%Ru/CNT catalyst. (Reaction conditions: catalyst—5%Ru/CNT; amount of catalyst—25 mg; temperature—200 °C; NH_3_·H_2_O—2.5 mL; H_2_ pressure—10 atm; lactic acid—5.6 mmol; ALA—alanine; LAM—lactamide; AA—acetic acid; PA—pyruvic acid).

The influences of reaction parameters, such as reaction time, temperature, and the amount of catalyst, were investigated. As shown in Table 4 and Figure 12, alanine selectivity increased with as the reaction time was elongated at high loads of lactic acid (i.e., 5.6 mmol). However, lactamide was still present in moderate amounts while high amounts of acetic acid were formed (Scheme 2).

Variable amounts of acetic acid were identified on both Ru/BEA and Ru/CNT (Table 5 and Figure 12) at an extended reaction time. This by-product could have originated from the oxidation of the aldehyde intermediate, formed through the decarbonylation/decarboxylation of the lactic and pyruvic acid (Scheme 2). The formation of acetic acid by-products could involve the contribution of the catalyst’s bifunctionality in a two-step process: the decarbonylation/decarboxylation reaction through C-C bond cleavage of lactic acid and/or pyruvic acid intermediate on ruthenium nanoparticles and/or acid sites followed by the subsequent hydrogenation of the resulting acetaldehyde on metallic ruthenium nanoparticles [46]. The higher amounts of acetic acid over Ru/BEA (Table 5) in comparison to those obtained over Ru/CNT catalysts (Figure 12) can be explained through the higher acidity of the former sample, as shown via NH_3_-TPD analysis (100 μmol/g, Figure 8 and Table S2, versus 2.2 μmol/g, Figure 4).

**Figure 13:**
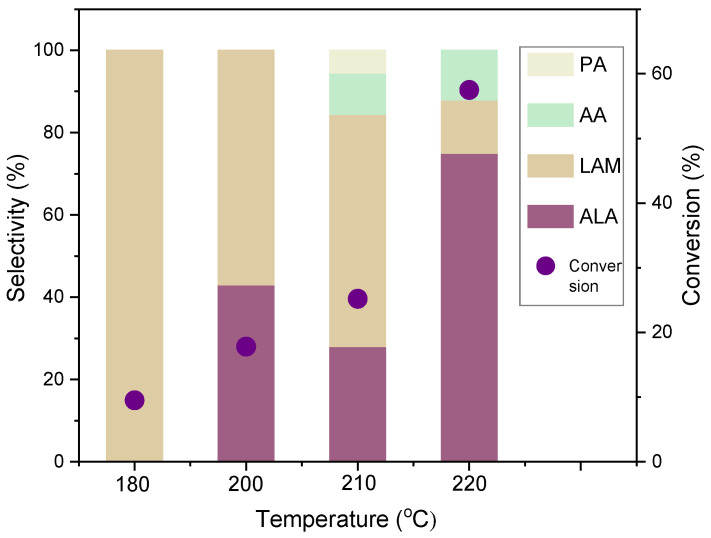
Distribution of the reaction products at different reaction temperatures in the presence of the 5%Ru/CNT catalyst. (Reaction conditions: lactic acid—0.7 mmol; catalyst—25 mg; NH_3_·H_2_O—2.5 mL; H_2_ pressure—10 atm; reaction time—2 h; ALA—alanine; LAM—lactamide; AA—acetic acid; PA—pyruvic acid).

**Figure 14:**
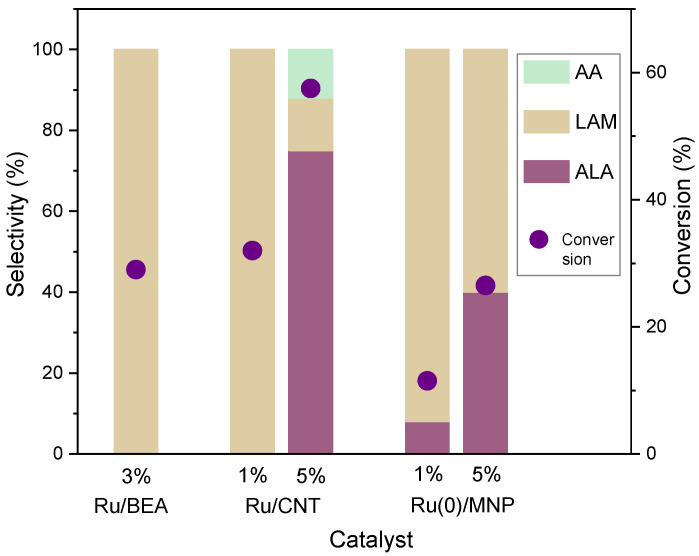
Distribution of the reaction products in the presence of different catalysts. (Reaction conditions: lactic acid—0.7 mmol; catalyst—25 mg; temperature—220 °C; NH_3_·H_2_O—2.5 mL; H_2_ pressure—10 atm; reaction time—2 h; ALA—alanine; LAM—lactamide; AA—acetic acid).

The effect of temperature was also studied for a load of lactic acid of 0.7 mmol. As Figure 13 and Figure 14 show, lactic acid conversion gradually increased when the temperature was elevated from 180 °C to 220 °C. The selectivity to alanine also showed temperature dependence. Therefore, at 220 °C, the selectivity to alanine reached 75.0% (yield of 45.0%) for a conversion of lactic acid of 60.0%, over the 5%Ru/CNT catalyst.

However, the catalytic efficiency was highly affected at a high temperature for lower loads of ruthenium (i.e., 1 and 3%), irrespective of the carrier nature (Figure 14).

**Figure 15:**
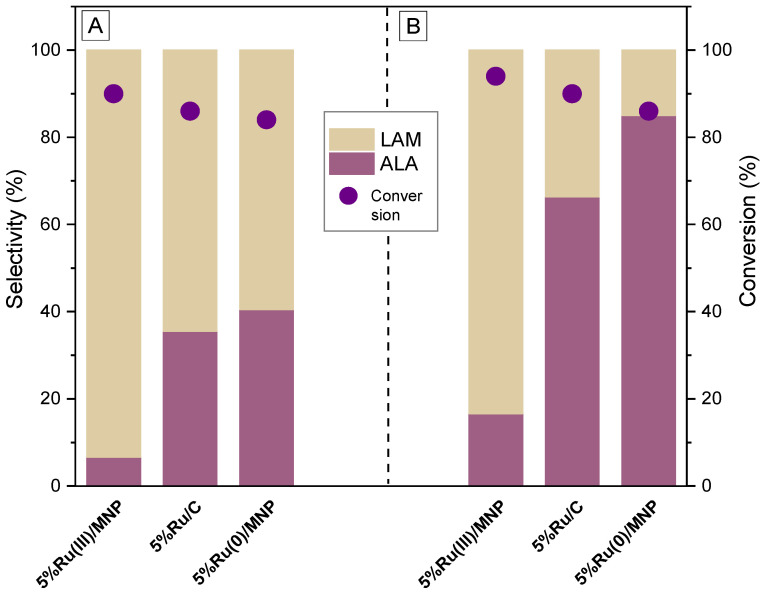
Catalytic results in terms of lactic acid conversion (C%) and selectivities (S%) to alanine (ALA) and lactamide (LAM) in the presence of 5%Ru(III)/MNP, 5%Ru/C, and 5%Ru(0)/MNP samples at 180 °C (**A**) and 200 °C (**B**). Reaction conditions: catalyst—50 mg; lactic acid—0.35 mmol; NH_3_·H_2_O—2.5 mL; H_2_ pressure—10 atm; time—2 h.

Finally, the influence of the catalyst amount was exemplified on the 5%Ru(0)/MNP catalyst at 180 °C and 200 °C (lactic acid load of 0.35 mmol); the obtained results are shown in Figure 15, showing a comparison to the results obtained on the 5%Ru(III)/MNP and commercial 5%Ru/C samples. Irrespective of the reaction temperature, the 5%Ru(0)/MNP catalyst achieved the highest catalytic performance, with a selectivity to alanine of 85%. The 5%Ru/C exhibited a selectivity to alanine of 62%, while 5%Ru(III)/MNP exhibited inferior catalytic performances with a selectivity to alanine of only 18%. The adjacent value of the catalytic effect between 5%Ru(III)/MNP, 5%Ru/C, and 5%Ru(0)/MNP implies that the highly dispersed metallic Ru nanoparticles are the main active sites of 5%Ru(0)/MNP. Moreover, the basic -NH_2_ groups promote the reaction whilst working together, facilitating the adsorption of acidic reactant.

The high efficiency of the 5%Ru(0)/MNP sample was also proven with a comparison of lactic acid amination to alanine on Ru-based catalysts of the obtained results in this work versus the results in the selected literature (Table 5). 

**Figure 16:**
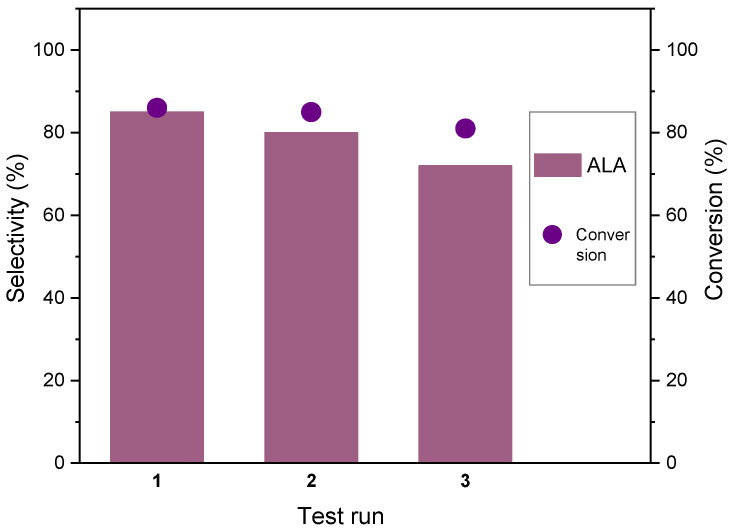
The recyclability experiments of 5%Ru(0)/MNP. Reaction conditions: catalyst—50 mg; lactic acid—0.35 mmol; NH_3_·H_2_O—3.5 mL; H_2_ pressure—10 atm; time—2 h.

The recyclability of the 5%Ru(0)/MNP catalyst was also evaluated in the conversion of lactic acid into alanine. Considering that the magnetic separation of the catalyst is more difficult from a small volume of a solvent, the volume of the NH_3_·H_2_O solution was increased to 3.5 mL (Figure 16) and was considered as optimal, allowing for easy catalyst separation. The catalyst could be used for at least three cycles without the catalytic efficiency being significantly modified in terms of lactic acid conversion and selectivity to alanine (Figure 16). The slight decrease in catalyst efficiency may be attributed to the inevitable loss of the reused catalyst during catalyst recovery.

**Figure 17:**
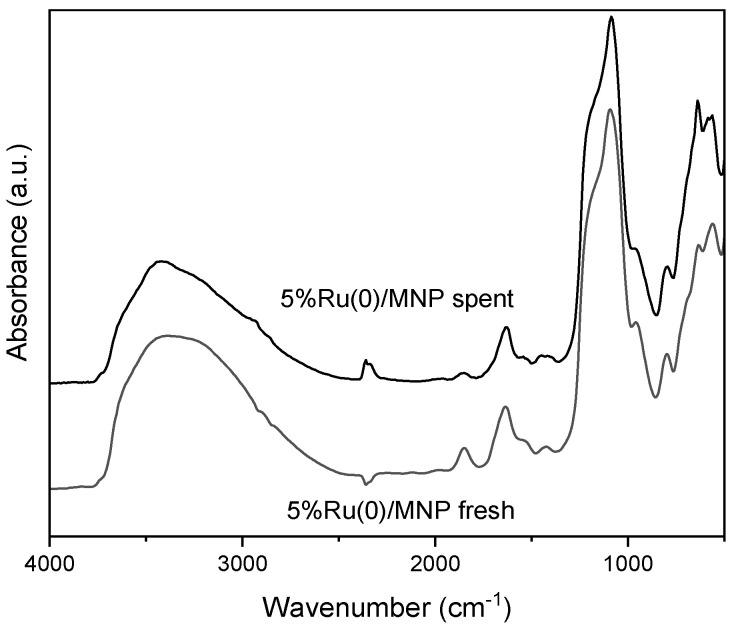
The DRIFT spectra of the fresh and spent 5%Ru(0)/MNP catalyst after the 3rd cycle.

The leaching test carried out showed that the 5%Ru(0)/MNP catalyst is stable: after the separation of the catalyst from the liquid solution, neither the conversion of lactic acid nor the product distribution were changed after another 1 h, and there was no leaching of the Ru species detected via ICP analysis for the reaction mixture. Therefore, it can be concluded that the catalyst is stable under the reaction conditions outlined and that the reaction takes place under heterogeneous conditions. Also, no significant changes of the existing functional groups were observed in the DRIFT spectra before the first catalytic cycle and after the third one (Figure 17). Moreover, the catalyst kept its magnetic character, being easily separated from the reaction products when an external magnet was applied.

However, after the 3rd catalytic cycle in the DRIFT spectra, new bands at 2200–2300 cm^−1^ were visible and were assigned to strongly chemisorbed organic molecules on the -NH_2_ sites. Thus, the decrease in the catalyst’s activity and yield to alanine were likely related to the blockage of the basic sites caused by organic molecules. However, drying the used catalyst in N_2_ flow at 100 °C, for 10 h, restored their initial activity and selectivity.

The catalytic efficiency in combination with the practical advantages of the catalyst (i.e., easy preparation of the catalyst and easy separation of the catalyst by applying an external magnetic field) make this catalytic system appealing for use in applications regarding the reductive amination of lactic acid to alanine.

## 4. Conclusions

A series of Ru-based catalysts with Ru loads of 1–5 wt% and MWCNT, BEA zeolite (Si/Al = 12.5), and MNP carriers were synthesized for the evaluation of lactic acid’s conversion to alanine. The characterization analysis results indicated that ruthenium could be dispersed uniformly on all carriers.

In relation to the Ru/CNT samples, ruthenium nanoparticles of different size and oxidation degrees were identified on the external side of the CNT tubes: the higher the ruthenium load, the larger the nanoparticles; the larger the nanoparticles, the higher the proportion of RuO_x_ (the lower the reduction degree). The high catalytic performance of the Ru-based catalysts over the Ru/CNT sample (selectivity to alanine of 87.0% for a conversion of lactic acid of 64.0%), comparable with those reported in the literature, can be explained by the existence of Ru/RuO_x_ couples on the outer surface of the CNT tubes—in which Ru promotes the dehydrogenation/hydrogenation steps and RuO_x_ activates carbonyl groups as Lewis acidic sites.

The characterization of the ruthenium-based BEA zeolite catalysts also indicated the presence of RuO_x_ nanoparticles and metallic Ru nanocrystallites, which were highly dispersed on the surface of the carrier. However, the presence of weak acid sites originated from the beta-zeolite structure, and highly influenced the products’ distribution by generating high amounts of acetic acid in the detriment of alanine selectivity. Hence, the Ru/BEA catalyst exhibited a moderate preference toward alanine, achieving a maximum selectivity of 27.0% for a lactic acid conversion of 24.5%. The obtained results can be correlated with the dissimilarities in catalytic characteristics (attributed to the catalyst preparation method), suggesting that the DP approach employed in this study yields significant variances when compared to the impregnation method described in the reported literature.

The catalytic performance of the Ru/MNP catalyst was found to be exceptional, exhibiting the highest selectivity to alanine at 85.0% for a conversion of lactic acid at 87.0%. This surpasses the performance of both the Ru/CNT and Ru/BEA catalysts, as well as many other catalysts previously reported in the literature. The key factor contributing to this remarkable efficiency is the presence of highly dispersed metallic Ru nanoparticles on the external surface of the catalyst. These nanoparticles serve as the primary active sites for the reaction, while the basic -NH_2_ groups play a cooperative role by facilitating the adsorption of the acidic reactant. This unique combination of active sites and cooperative promotion enables the catalyst to achieve such impressive results. Additionally, the practical advantages of this catalytic system, such as its easy preparation and the ability to separate the catalyst using an external magnetic field, further enhance its appeal for applications in the reductive amination of lactic acid to alanine.

## Figures and Tables

**Table 1:** The pore structure parameters of Ru/CNT samples.

Entry	Sample	S_BET_ (m^2^/g)	Micropore Surface Area (m^2^/g)	External Surface Area (m^2^/g)	Total pore Volume (cm^3^/g)	Micropore Volume (cm^3^/g)	Mezopores Volume (cm^3^/g)	Average Pore Size (BJH, nm)
1	1% Ru/CNT	584	56	527	3.82	0.02	3.80	3.3 and 27.7
2	3% Ru/CNT	315	22	293	1.67	0.01	1.66	3.3 and 31.9
3	5% Ru/CNT	310	20	291	1.88	0.01	1.87	3.3 and 30.5

**Table 2:** Textural properties of the pristine BEA zeolite and Ru/BEA12.5 samples.

Entry	Sample	S_BET_ (m^2^/g) ^a^	S_ext_ (m^2^/g) ^b^	S _micro_ (m^2^/g) ^b^	V _total_ (cm^3^/g) ^c^	V_meso_ (cm^3^/g) ^d^	V_micro_ (cm^3^/g) ^e^	V_micro_/ V_meso_	Avarage Pore size (nm) ^f^
1	BEA	495	186	309	0.72	0.58	0.14	0.24	8.5; 31.7
2	1%Ru/BEA	415	168	247	0.65	0.54	0.11	0.20	9.3; 30.0
3	3%Ru/BEA	402	186	216	0.44	0.37	0.07	0.19	9.3; 28.3

^a^—calculated using the BET method; ^b^—external surface area calculated using the t-plot method; ^c^—the total pore volume determined at a relative pressure (p/p_0_) of 0.98; ^d^—the mesopores volume calculated using the BJH method; ^e^—the micropores volume calculated using the t-plot method; ^f^—mesopore diameter calculated using the BJH method.

**Table 3:** Catalytic results in terms of lactic acid conversion (C%) and selectivities (S%)/yields (%) to alanine (ALA) and lactamide (LAM) as a function of lactic acid loads.

Entry	Catalyst	LA mmol	C (%)	S(%)	
				ALA	LAM
1	5%Ru/CNT	0.35	64.0	87.0	13.0
2	5%Ru(0)/MNP	0.35	85.0	83.0	17.0
3	1%Ru/BEA	0.7	4.7	-	100
4	3%Ru/BEA	0.7	18.2	-	100
5	1%Ru/CNT	0.7	10.3	-	100
6	3%Ru/CNT	0.7	8.8	-	100
7	5%Ru/CNT	0.7	17.8	43.0	57.0
8	1%Ru(0)/MNP	0.7	11.0	10.0	90.0
9	5%Ru(0)/MNP	0.7	40.5	11.3	88.7
10	1%Ru/BEA	5.6	5.0	-	100
11	3%Ru/BEA	5.6	6.3	-	100
12	1%Ru/CNT	5.6	13.5	5.0	95.0
13	5%Ru/CNT	5.6	17.5	7.5	92.5
14	1%Ru(0)/MNP	5.6	11.6	5.3	96.7
15	5%Ru(0)/MNP	5.6	31.6	17.5	82.5

Reaction conditions: catalyst—25 mg; NH_3_·H_2_O—2.5 mL; temperature—200 °C; H_2_ pressure—10 atm; time—2 h; ALA—alanine; LAM—lactamide.

**Table 4:** Catalytic performances of Ru/BEA catalysts in terms of lactic acid conversion (C%) and selectivities (S%) to alanine (ALA) as a function of the reaction time.

Entry	Catalyst	Reaction Time (h)	C (%)	S(%)		
				ALA	LAM	AA
1	1%Ru/BEA	4	13.5	5.1	20.9	74.0
2	1%Ru/BEA	8	17.0	12.0	29.0	59.0
3	3%Ru/BEA	4	22.2	5.5	58.5	36.0
4	3%Ru/BEA	8	24.5	27.0	62.0	11.0

Reaction conditions: catalyst—25 mg; lactic acid—5.6 mmol; NH_3_·H_2_O—2.5 mL; temperature—200 °C; H_2_ pressure—10 atm; ALA—alanine; LAM—lactamide; AA—acetic acid.

**Table 5:** Selected literature results of lactic acid amination to alanine on Ru-based solid catalysts.

Entry	Catalyst	LA (mmol)	Time (h)	T (°C)	P_H2_ (bar)	C%	S%	Ref.
1	Ru/H-β (Si/Al = 12.5)	2.0	4	220	10	55.0	80.0	[15]
2	Ru/C	2.0	4	220	10	60.0	80.0	[15]
3	Ru/CNT	2.0	4	220	10	83.0	57.0	[15]
4	Ru/TiO_2_	50 mg PLA	72	140	-	>99	70.0	[12]
5	Ru/TiO_2_	50 mg PLA	18	140	10	>99	27.0	[12]
6	Ru/Ni@C	1.11	2	220	15	70.5	75.0	[16]
7	Ru/CS	1.11	2	220	15	63.4	77.5	[16]
8	Ru/CNT	0.5	2	220	10	75.0	49.0	[5]
9	Ru_1_Ni_7_MgO	1.11 (glycerol)	1	220	10	15.0	0.08	[8]
10	Ru/N-CNTs	0.5	2	180	10	99.9	70.0	[11]
11	Ru/ZSM-5	0.5	2	180	10	69.0	0	[11]
12	Ru/CNTs	0.5	2	180	10	94.5	53.9	[11]
13	Ru/CNT	0.35	2	200	10	64.0	87.0	This work
14	Ru/CNT	0.7	2	220	10	60.0	75.0	This work
14	Ru/BEA	5.6	8	200	10	24.5	27.0	This work
15	Ru/MNP	0.35	2	200	10	87.0	85.0	This work

## Data Availability

Data are contained within this article or the Supplementary Materials.

## Supplementary Materials

The following supporting information can be downloaded at: https://www.mdpi.com/article/10.3390/nano14030277/s1, Figure S1: GC chromatograms obtained at different temperatures after derivatization with MTBSTFA for 4 h. In figure is shown the peak for the derivatized standard of alanine (inset) and the derivatized alanine identified in the reaction in the following conditions: catalyst 5%Ru(III)/MNP—50 mg; lactic acid—0.35mmol; temperature—200 °C; NH_3_·H_2_O—2.5 mL; H_2_ pressure—10 atm; reaction time—2 h; Figure S2: GC-MS identification of alanine: Mass spectra for alanine obtained in the reaction mixture compared to the database; Figure S3: Calibration curve and the corresponding trendline equation for the analysis of alanine solutions in NH_3·_H_2_O using UV-Vis spectrophotometry. The sample obtained after reaction under following reaction conditions: catalyst 5%Ru(0)/MSN—50 mg; lactic acid—0.35 mmol; temperature—200 °C; NH_3_·H_2_O—2.5 mL; H_2_ pressure—10 atm; reaction time—2 h; Figure S4: Representative GC chromatogram of the reaction products obtained in the following conditions: 5%Ru/CNT—25 mg; lactic acid—0.7 mmol; temperature—210 °C; NH_3_·H_2_O—2.5 mL; H_2_ pressure—10 atm; reaction time—2 h. Where: 1-acetic acid (AA); 2-pyruvic acid (PA); 3-lactic acid (LA); 4-alanine (AL); 5-lactamide (LAM).Figure S5: Nitrogen adsorption-desorption isotherm and pore size distributions (inset) for 1%Ru/CNT sample; Figure S6: Nitrogen adsorption-desorption isotherm and pore size distributions (inset) for 3%Ru/CNT sample; Figure S7: Nitrogen adsorption-desorption isotherm and pore size distributions (inset) for 5%Ru/CNT sample; Figure S8: BF and DF-STEM images for the 3%Ru/CNT sample; Figure S9: BF-STEM images for the 5%Ru/CNT sample: Figure S10: H2-TPD profiles for the Ru/CNT samples: Figure S11: Adsorption-desorption isotherm of liquid nitrogen at −196 °C of BEA (A), 1%Ru/BEA (B) and 3%Ru/BEA (C) sample. Inset: Pore size distribution; Table S1: H2-TPD parameters for Ru/CNT samples and MWCNT carrier; Table S2: Acidic characteristics of the pristine BEA zeolite and 3%Ru/BEA catalyst, determined from the NH_3_-TPD; Scheme S1: Derivatization of aminoacids with MTBSTFA agent.
