# Supplementary material for: Highly Efficient Ru-Based Catalysts for Lactic Acid Conversion to Alanine
Source: Nanomaterials (Basel). 2024 Jan 29;14(3):277. doi: 10.3390/nano14030277 (PMC10856861; doi:10.3390/nano14030277)
Supplement: Supplementary file 1 [file nanomaterials-14-00277-s001.zip › nanomaterials-2816554-supplementary.pdf]

Supplementary file

Article

Highly Efficient Ru-Based Catalysts for Lactic Acid Conversion to Alanine

Iunia Podolean [1], Mara Dogaru [1], Nicolae Cristian Guzo [1], Oana Adriana Petcuta [1], Elisabeth E. Jacobsen [2],
Adela Nicolaev [3], Bogdan Cojocaru [1], Madalina Tudorache [1], Vasile I. Parvulescu [1] and Simona M. Coman [1,*]

[1] Department of Inorganic Chemistry, Organic Chemistry, Biochemistry and Catalysis, Faculty of Chemistry,
University of Bucharest, 4-12 Regina Elisabeta Av., 030018 Bucharest, Romania; iunia.podolean@chimie.unibuc.ro (I.P.); mara.badea@s.unibuc.ro (M.D.); nicolae.guzo@s.unibuc.ro (N.C.G.); oana.petcuta@s.unibuc.ro (O.A.P.); bogdan.cojocaru@chimie.unibuc.ro (B.C.); madalina.sandulescu@chimie.unibuc.ro (M.T.); vasile.parvulescu@chimie.unibuc.ro (V.I.P.)
[2] Department of Chemistry, Norwegian University of Science and Technology, Høgskoleringen 5,
7491 Trondheim, Norway; elisabeth.e.jacobsen@ntnu.no
[3] National Institute of Materials Physics, Atomistilor 405b, 077125 Magurele, Ilfov, Romania; adela.nicolaev@infim.ro
* Correspondence: simona.coman@chimie.unibuc.ro

Product analysis

Typically, the HPLC analysis of the reaction mixture containing amino acids is a complex one, performed with two different columns and a complex elution system, and requires derivatization with o-phthalaldehyde, the obtained derivatives being unstable. For this reason, the GC-MS method was chosen for the analysis of the products, which allows simultaneous separation and identification. Two commercial derivatizing agents, namely N,O-Bis(trimethylsilyl)trifluoroacetamide:trimethylchlorosilane 99:1 (BSTFA) and N-Methyl-N-tert-butyldimethylsilyltrifluoroacetamide (MTBSTFA) were tested. During the derivatization reaction, the active hydrogen in -OH and $-NH_2$ groups is replaced by silane groups (for exemplification see Scheme S1). Among the two, MTBSTFA showed superior ability for alanine silylation and much better stability, which is also in agreement with the literature.

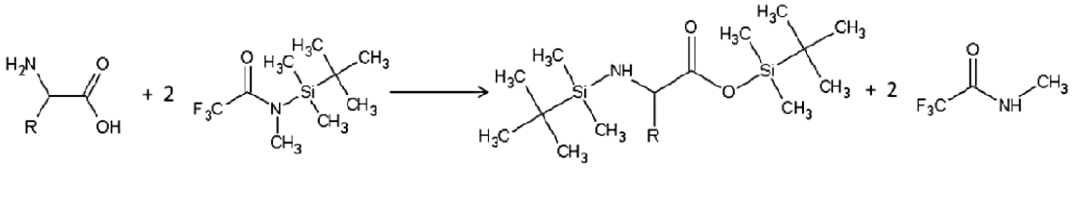

Scheme S1. Derivatization of aminoacids with MTBSTFA agent

High temperatures, often in the presence of acetonitrile, are required for complete derivatization of amino acids. Several derivatization temperatures were tested and the results are shown in Figure 1S. A complete dissolution of the reaction mixture and a higher relative abundance (%) were observed at 80 and 90 °C, respectively. However, no major differences were observed between the silylation results at 80 and 90 °C. Therefore, the derivatization was performed at 80 °C in order to avoid the formation of secondary derivatives.

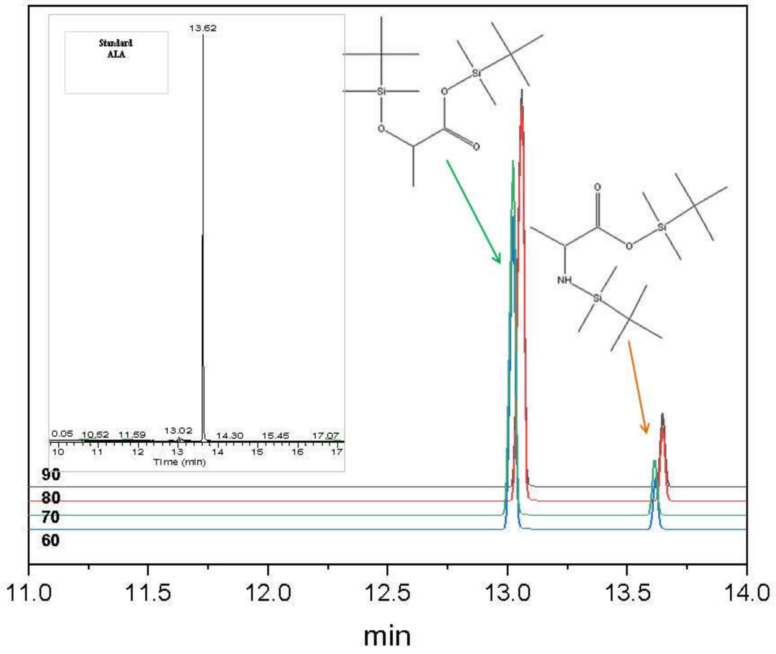

Figure S1. GC chromatograms obtained at different temperatures after derivatization with MTBSTFA for 4h. In figure is shown the peak for the derivatized standard of alanine (inset) and the derivatized alanine identified in the reaction in the following conditions*: catalyst 5%Ru(III)/MNP - 50mg; lactic acid-0.35mmol; temperature-200ºC;* $NH_3 \cdot H_2O$*-2.5mL;* H_2 *pressure-10atm; reaction time- 2h.*

The mass spectra of the corresponding peaks were identified using the MS database, but also by injecting the derivatized standard compound under the same conditions. Figure S2 shows the mass spectra for the alanine peak in the reaction mixture compared to that in the database, which are identical at a probability of 93%.

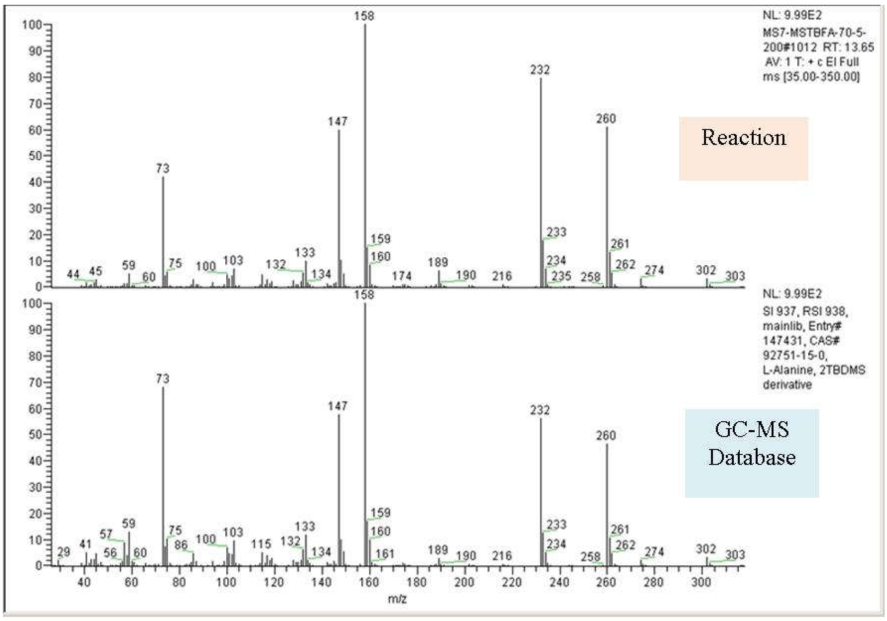

Figure S2. GC-MS identification of alanine: Mass spectra for alanine obtained in the reaction mixture compared to the database.

For additional confirmation, a UV-Vis spectrophotometric analysis of a reaction mixture was also performed using a calibration curve of alanine in $NH_3{\cdot}H_2O$ with ninhydrin. The calibration curve and the corresponding trendline are shown in Figure S3.

The concentration determined for sample was 0.158 mmol/mL, corresponding to 0.395 mmol in 2.5 mL and respectively a yield of 82%, comparable to that obtained by GC-MS (85%).

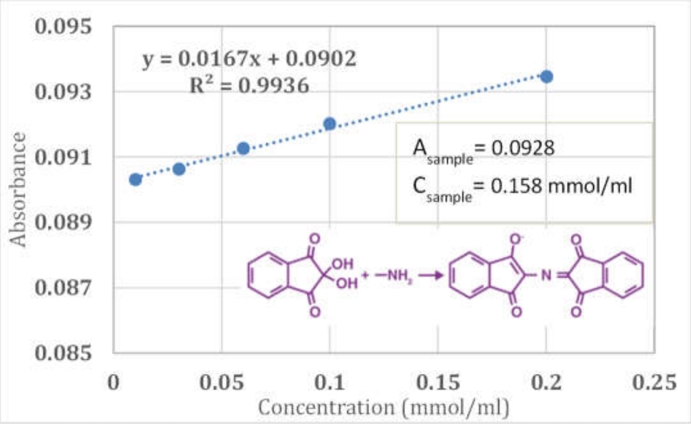

Figure S3. Calibration curve and the corresponding trendline equation for the analysis of alanine solutions in $NH_3 \cdot H_2O$ using UV-Vis spectrophotometry. The sample obtained after reaction under following reaction conditions: *catalyst 5%Ru(0)/MSN - 50mg; lactic acid-0.35mmol; temperature-200ºC;* $NH_3 \cdot H_2O$ *-2.5mL; H_2 pressure-10atm; reaction time- 2h*

To confirm the successful separation and identification of all reaction products, a complete GC chromatogram of a reaction mixture is provided in Figure S4.

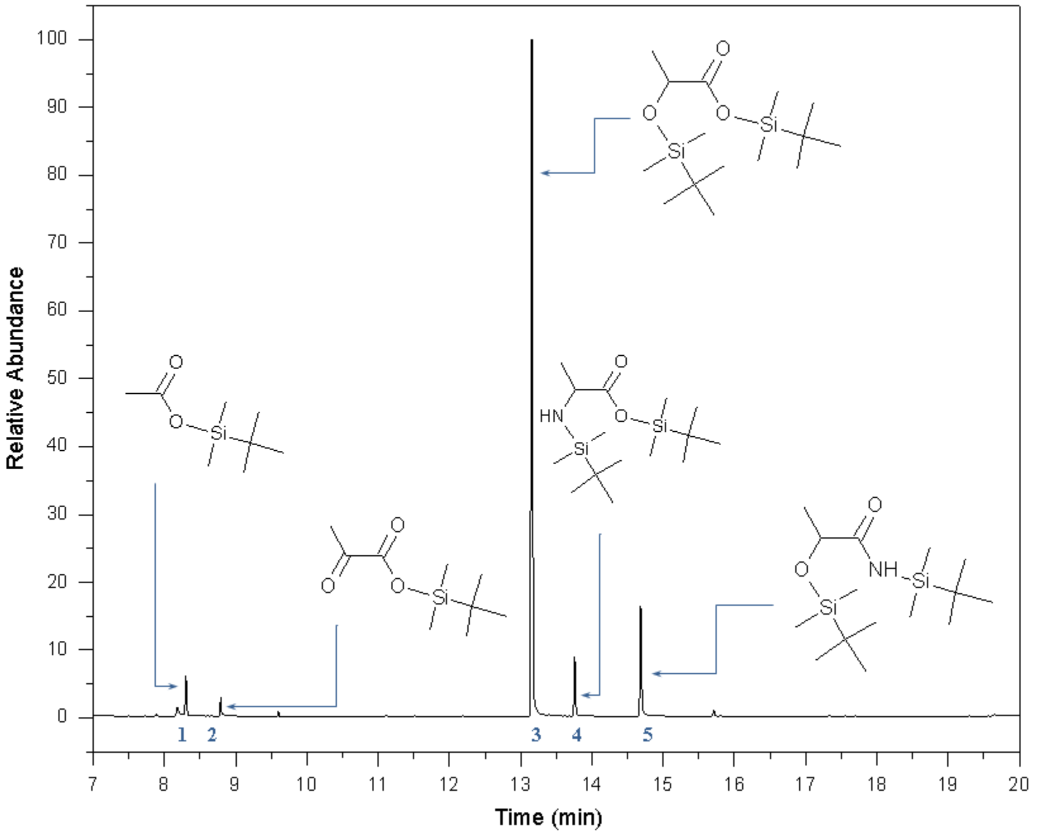

Figure S4. Representative GC chromatogram of the reaction products obtained in the following conditions: *5%Ru/CNT-25mg; lactic acid-0.7mmol; temperature-210°C;* $NH_3{\cdot}H_2O$ *-2.5mL;* H_2 *pressure-10atm; reaction time- 2h*. Where: 1-acetic acid (AA); 2- pyruvic acid (PA); 3 - lactic acid (LA); 4-alanine (AL); 5-lactamide (LAM).

Liquid nitrogen adsorption-desorption isotherms at -196 °C of Ru/CNT samples

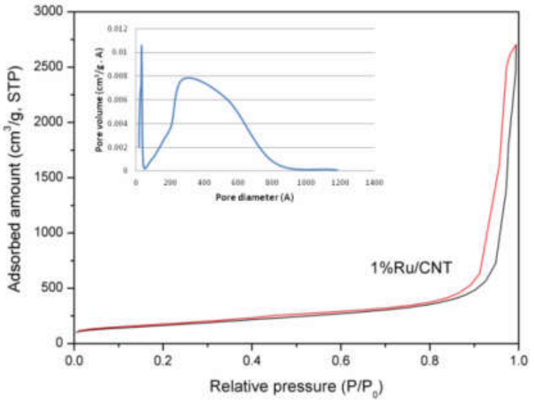

Figure S5. Nitrogen adsorption-desorption isotherm and pore size distributions (inset) for 1%Ru/CNT sample

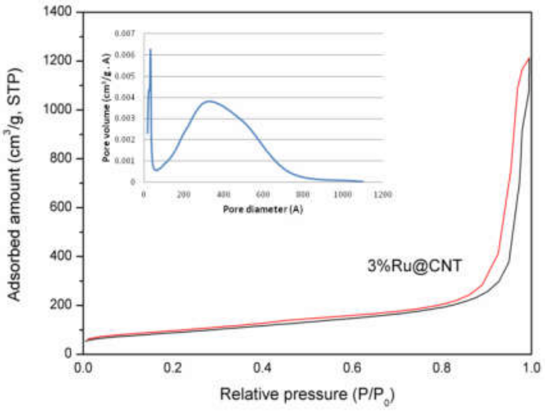

Figure S6. Nitrogen adsorption-desorption isotherm and pore size distributions (inset) for 3%Ru/CNT sample

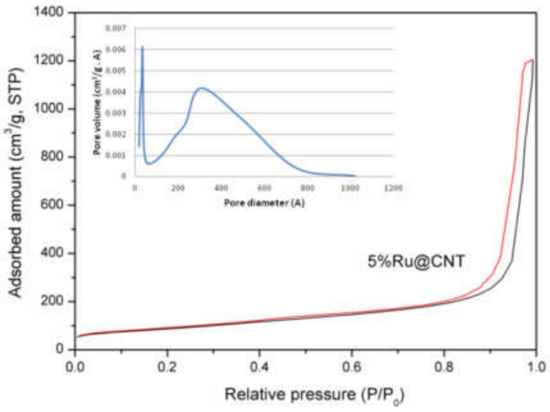

Figure S7. Nitrogen adsorption-desorption isotherm and pore size distributions (inset) for 5%Ru/CNT sample

STEM microscopy

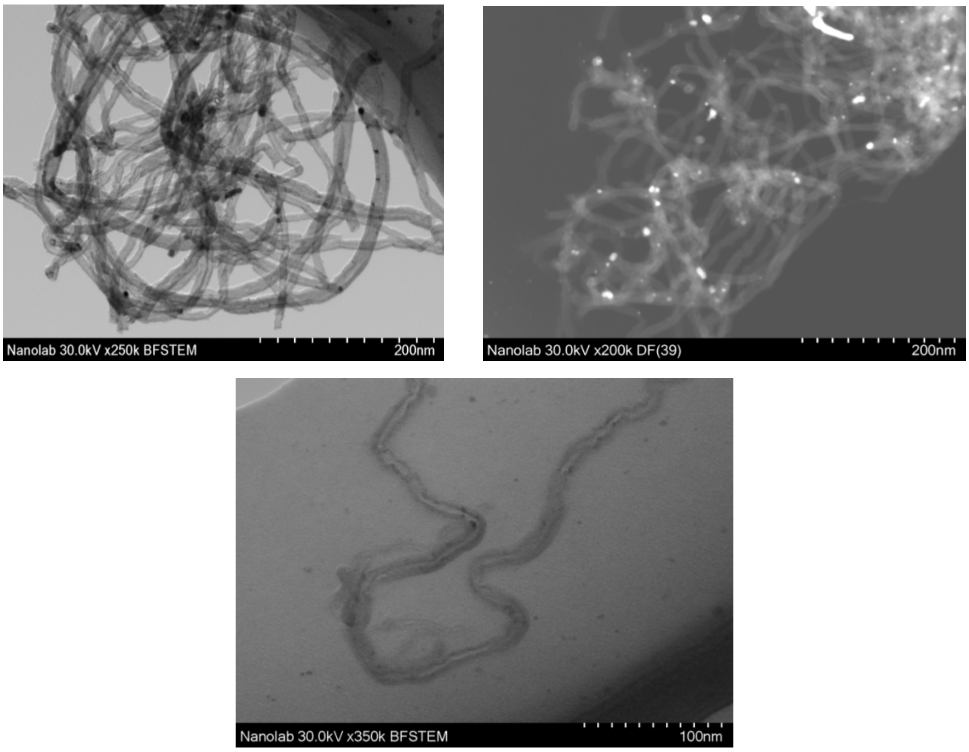

Figure S8. BF and DF-STEM images for the 3%Ru/CNT sample

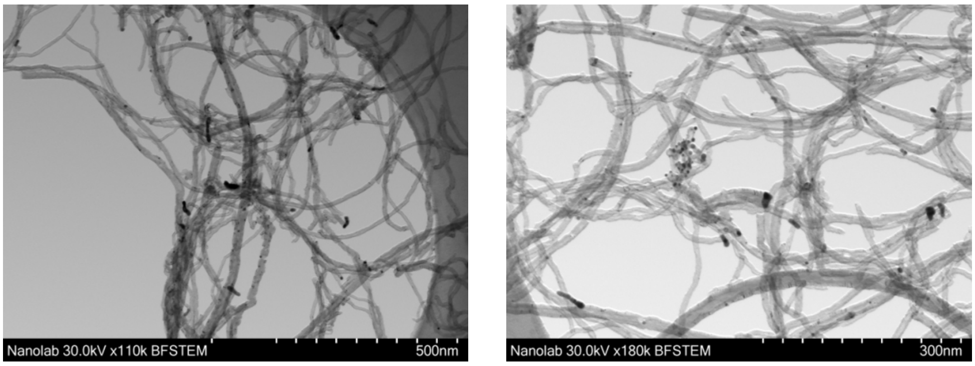

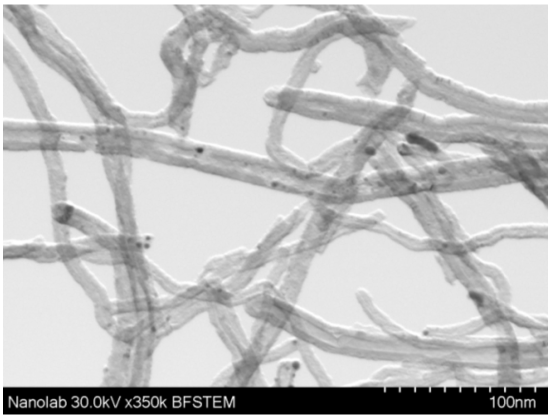

Figure S9. BF-STEM images for the 5%Ru/CNT sample

H_2-TPD

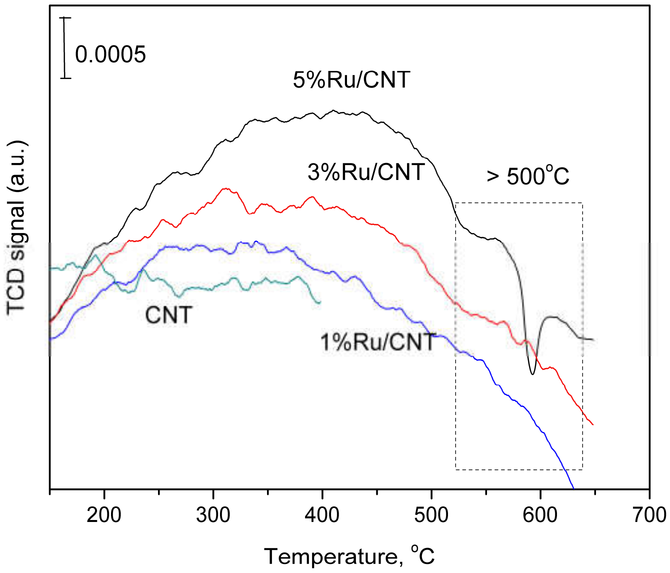

Figure S10. H_2-TPD profiles for the Ru/CNT samples

Table S1. H_2-TPD parameters for Ru/CNT samples and MWCNT carrier

Entry	Sample	Temperature at Maximum (°C)	H_2 desorbed (mmol/g)
1	CNT	-	0

2	1%Ru/CNT	324	0.007
		338	0.002
		430	0.007
		489	0.010
		**TOTAL**	**0.026**
3	3%Ru/CNT	311	0.017
		565	0.015
		587	0.003
		610	0.003
		**TOTAL**	**0.038**
4	5%Ru/CNT	306	0.035
		561	0.005
		609	0.006
		**TOTAL**	**0.046**

NH_3- TPD results for Ru/BEA catalysts

Table S2. Acidic characteristics of the pristine BEA zeolite and 3%Ru/BEA catalyst, determined from the NH_3- TPD.

Sample	Range of temperature (°C)				Total sites conc. (µmols /g)
	50-100	100-200	200-300	300-400	
BEA zeolite	17.8	80.5	-	10.2	108.5
3%Ru/BEA	-	65.8	34.2	-	100.0

Liquid nitrogen adsorption-desorption isotherms at -196 °C of Ru/BEA samples

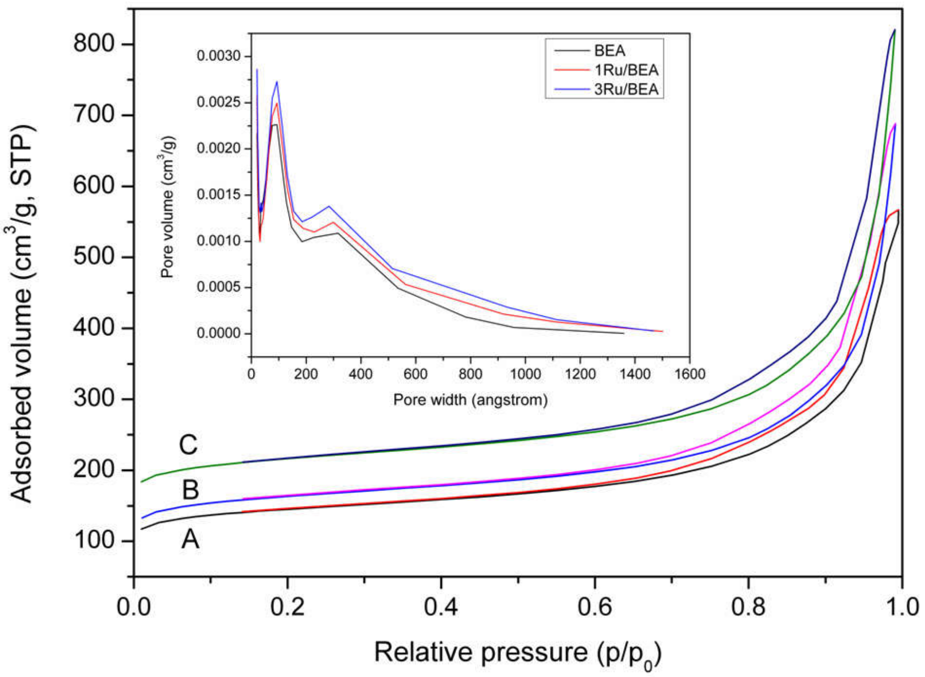

Figure S11. Adsorption-desorption isotherm of liquid nitrogen at -196°C of BEA (A), 1%Ru/BEA (B) and 3%Ru/BEA (C) sample. Inset: Pore size distribution
